# Prevalence of HIV-associated osteoporosis and fracture risk in midlife women: a cross-sectional study in Zimbabwe

**DOI:** 10.1093/jbmr/zjae138

**Published:** 2024-07-09

**Authors:** Tafadzwa Madanhire, Mícheál Ó Breasail, Cynthia Kahari, Farirayi Kowo-Nyakoko, Peter R Ebeling, Rashida A Ferrand, Kate A Ward, Celia L Gregson

**Affiliations:** The Health Research Unit Zimbabwe, Biomedical Research and Training Institute, Harare, Zimbabwe; Infectious Disease Epidemiology, Faculty of Epidemiology and Population Health, London School of Hygiene and Tropical Medicine, London WC1E 7HT, United Kingdom; Department of Medicine, School of Clinical Sciences, Faculty of Medicine, Monash Medical Centre, Nursing and Health Sciences, Monash University, Clayton VIC 3168, Australia; Population Health Sciences, Bristol Medical School, Bristol BS8 1NU, United Kingdom; The Health Research Unit Zimbabwe, Biomedical Research and Training Institute, Harare, Zimbabwe; Infectious Disease Epidemiology, Faculty of Epidemiology and Population Health, London School of Hygiene and Tropical Medicine, London WC1E 7HT, United Kingdom; The Health Research Unit Zimbabwe, Biomedical Research and Training Institute, Harare, Zimbabwe; MRC Lifecourse Epidemiology Centre, Human Development and Health, University of Southampton, Southampton SO16 6YD, United Kingdom; Department of Medicine, School of Clinical Sciences, Faculty of Medicine, Monash Medical Centre, Nursing and Health Sciences, Monash University, Clayton VIC 3168, Australia; The Health Research Unit Zimbabwe, Biomedical Research and Training Institute, Harare, Zimbabwe; Clinical Research Department, Faculty of Infectious and Tropical Diseases, London School of Hygiene and Tropical Medicine, London WC1E 7HT, United Kingdom; MRC Lifecourse Epidemiology Centre, Human Development and Health, University of Southampton, Southampton SO16 6YD, United Kingdom; MRC Unit, The Gambia @ London School of Hygiene and Tropical Medicine, Banjul, The Gambia; The Health Research Unit Zimbabwe, Biomedical Research and Training Institute, Harare, Zimbabwe; Global Musculoskeletal Research Group, Musculoskeletal Research Unit, Bristol Medical School, University of Bristol, Bristol BS10 5NB, United Kingdom

**Keywords:** HIV, BMD, DXA, menopause, sub-Saharan Africa, fracture, FRAX

## Abstract

Antiretroviral therapy roll-out has dramatically reduced HIV-related mortality; more women are living to reach menopause. Menopausal estrogen loss causes bone loss, as does HIV and some of its treatments. However, data describing HIV’s impact on osteoporosis prevalence and fracture risk are scarce in southern Africa. A cross-sectional study of women aged 40-60 years (49% women with HIV [WLH]) was conducted in Harare, Zimbabwe. Menopause, fracture, and HIV history were collected, and anthropometry and BMD (by DXA) measured, and FRAX 10-year fracture probabilities quantified. The FRAX probability of a major osteoporotic fracture (MOF) included HIV as a risk factor for secondary osteoporosis. Linear and Poisson regression determined the relationships between clinical risk factors and both femoral neck (FN) BMD and the 10-year FRAX probability of MOF respectively. The 393 participants had a mean (SD) age of 49.6 (5.8) years and mean (SD) BMI of 29.1 (6.0) kg/m^2^. 95% of WLH were antiretroviral therapy (ART) established (85% tenofovir disoproxil fumarate) and 81% had a viral load <50 copies/mL. A BMD T-score ≤ –2.5 was more common in WLH than those without, at both FN and lumbar spine (LS) (FN, 22 [11.4%] vs 5 [2.5%]; LS, 40 [20.8%] vs 9 [4.5%], respectively). Prior fracture was more prevalent in WLH: any fracture type (27 [14%] vs 14 [7%]); MOF (14 [7.3%] vs 5 [2.5%]). WLH had a higher 10-year MOF probability (median, 1.2%; IQR, 0.9-1.8) compared with those without HIV (1.0%; IQR, 0.9-1.5) (*p* < .001), although probabilities were low. Older age, low weight, and HIV infection were strongly associated with lower FN BMD. Higher probability of MOF was associated with older age, HIV infection, parental hip fracture and prior fracture, although adjustment attenuated the association with HIV. No woman reported anti-osteoporosis medication use. While osteoporosis and previous fractures were common and untreated in this relatively young population, particularly in WLH, the FRAX-predicted 10-year MOF risk was low. Clinical risk factors considered in fracture risk prediction tools in Zimbabwe may need contextual modification.

## Introduction

Two-thirds of the 38.4 million people with HIV in the world live in east and southern Africa.^1^ Here, the incidence of HIV disproportionately impacts women; in 2021, women and girls accounted for 63% of all new HIV infections.^2^ The widespread availability of antiretroviral therapy (ART) has markedly reduced HIV-related mortality,^3^ turning HIV into a chronic manageable, albeit incurable, condition. As life expectancy has increased, age-associated noninfectious comorbidities, such as osteoporosis, are becoming more prevalent among cohorts of people with HIV.^4–6^ Independent of age, HIV infection and its treatment may adversely affect bone health in women, even prior to menopause.^5–7^ Thus, it is probable that women living with HIV enter menopause with pre-existing bone deficits and/or may suffer greater menopause-related bone losses due to additional risk factors challenging bone health. 

In east and southern Africa, data on bone health in menopausal women are limited, with most available literature drawn from populations in South Africa, an upper-middle-income country.^6^^,^^8–11^ Of these South African studies, few have included a similar proportion of women living with HIV as seen nationally, where female prevalence is 23.5% in those aged 15–49 years.^12^ These studies suggest that women living with HIV may be more vulnerable to menopause-associated bone loss. Cross-sectional data from Soweto suggest that women living with HIV may have low bone mass and pre-existing compartment-specific bone deficits before menopause transition, yet experience broadly similar patterns of bone loss to HIV-negative women^12^^,^^13^; a longitudinal study from the same cohort provided stronger evidence of greater menopause-associated bone loss in women ageing with HIV.^14^ How this translates to fracture risk is unclear.

One major barrier to bone health assessment across the region is lack of access to bone densitometry. This inequity can potentially be mitigated, in part, by well-calibrated, population-specific fracture-prediction tools that do not depend upon BMD assessment. Recently, hip fracture rates in South Africa^15^ were used to produce ethnicity-specific FRAX models to facilitate fracture risk assessment.^16^ In 2022, these data enabled the calibration of a new “proxy” FRAX tool for Zimbabwe, providing the first opportunity to risk stratify patients in Zimbabwe (which has 3 DXA scanners among 15.2 million people). However, FRAX has not yet been validated in this setting. Previous research in other populations has suggested that FRAX may underestimate fracture risk in those living with HIV.^17^^,^^18^ The current study aimed to determine the prevalence of osteoporosis in midlife women living with and without HIV, the prevalence of prior fracture, and the estimated probabilities of future fracture using the new proxy FRAX tool in Zimbabwe.

## Materials and methods

### Study population

Between April and December 2020, women aged 40–60 years residing in Harare, Zimbabwe, were enrolled in a cross-sectional study, using age-stratified sampling to reflect 4 age groups (40–44, 45–49, 50–54, and 55–60 years to span the period of menopausal transition) and by HIV status (and established on ART), aiming to recruit 200 women with and 200 without HIV. The study recruited women attending for routine care at the HIV clinic at Sally Mugabe (formerly known as Harare Central) and Parirenyatwa hospitals, the 2 main public sector tertiary health facilities in Harare.

Due to the COVID-19 pandemic and the resulting lockdown in Zimbabwe, the original plan to recruit women without HIV from local churches in Harare was amended, as recruiting from church gatherings was restricted and recruitment was therefore unfeasible. An alternative approach was used whereby recruited women with HIV were asked to identify 2 female friends of a similar age with phone access who might be interested in participating. The recruited women had to be aged between 40 and 60 years, residing in Harare, not acutely unwell, and be willing to participate, including having an HIV test.

### Data collection

A female nurse–administered questionnaire collected data on sociodemographic characteristics (education attained, employment status, household income, and food insecurity [using selected questions from the US adult food security survey module^19^]), lifestyle information (smoking and alcohol intake), parity, and medical and prior fracture history by self-report (major osteoporotic fracture [MOF] [ie, hip, spine, wrist, and humerus] and fragility fractures [ie, resulting from a fall from standing height or less]). Socioeconomic status (SES) quintiles were estimated using principal components analysis of the participant’s household assets—(1) own dwelling, (2) electricity, (3) bicycle, (4) television, (5) working car/truck, (6) tap in house, (7) private water/borehole (running water), (8) flush toilet, (9) pit latrine, (10) solar energy, (11) household power supply, (12) washing machine, (13) power generator, and (14) internet access by computer (WiFi).^20^ Women currently having regular periods were classified as premenopausal, women having irregular periods were classified as perimenopausal, and women who had had no bleeding for more than 12 months were classified as postmenopausal. Twenty-one women reported a hysterectomy, 20 of whom also had oophorectomy. Hysterectomy prevents menopausal staging using menstrual bleeding criteria.^21^^,^^22^ Estrogen, a hormone with both anabolic and antiresorptive effects on bone,^23^ production declines following menopausal transition, while following oophorectomy, it ceases abruptly.^24^ Therefore, as described previously,^14^ the few women who reported a hysterectomy +/− oophorectomy within the last 12 months were added to the perimenopausal group (*n* = 1) (as their skeleton had experienced only 1 year or less of estrogen loss), and if they reported a hysterectomy more than 1 year ago, they were added to the postmenopausal group (*n* = 20), with preplanned sensitivity analyses (see later). Medications and dietary supplement use were recorded, including ART, estrogen replacement therapy, current contraceptive use, any bone health medicines, and calcium and/or vitamin D supplements. Anthropometry, DXA measurements, and HIV testing were performed, as described below.

### Anthropometry

Two nurses measured height (in cm) and weight (in kg), without shoes in light clothing, in triplicate using a Seca 213 stadiometer and Seca 875 digital scales (Seca Precision for Health, Seca Mechanical Floor Scales Class III, Hamburg, Germany), respectively, with mean weight or height calculated. Body mass index (BMI) was calculated (kg/m^2^) and categorized as underweight (<18.5 kg/m^2^), normal weight (18.5–24.9 kg/m^2^), overweight (25–29.9 kg/m^2^), and obese (≥30 kg/m^2^).^25^

### HIV testing

All women recruited without an established HIV diagnosis had a point-of-care HIV antibody test performed using the Alere Determine HIV-1/2 (Alere San Diego, Inc, San Diego, CA, USA). If negative, they were enrolled into the HIV-negative group. If positive, after a confirmatory test (Chembio SURE CHECK HIV 1/2 Assay), they were enrolled into the HIV-positive group (*n* = 1) and referred to local HIV services.^26^

### Blood tests

Blood samples (4 mL) were collected in EDTA tubes from which HIV viral load testing was performed using the Roche COBAS Ampliprep/COBAS Taqman 48.

### DXA

DXA scans were performed using the Hologic Discovery Wi instrument (Hologic, Inc, Bedford, MA, USA; Apex Version 13.4.2:3 software; S/N 83145); scanner performance over the period of the study was monitored using standard manufacturer quality-assurance and quality-control protocols. The scanner was operated by 1 trained radiographer who measured areal BMD of the total body (TB), lumbar spine (LS) (L1–L4), total hip (TH), and femoral neck (FN). Coefficients of variation for BMD DXA measurements, repeated twice in 30 participants, were 1.22%, 1.76%, 1.81%, and 1.75% for TB, LS, TH, and FN regions, respectively. T-scores were calculated using reference data from the National Health and Nutrition Examination Survey (NHANES) III as recommended by the International Society for Clinical Densitometry (ISCD), classifying a low T-score as less than or equal to –2.5.^27^

### Fracture risk assessment using FRAX

The study estimated the 10-year probability of an MOF using the 2022 Zimbabwean FRAX tool, calibrated using Black South African hip fracture incidence data.^28^^,^^29^ The exposures in the FRAX probability model are participant’s age, sex, ethnicity (country of origin), history of prior fracture parental hip fracture, current smoking, alcohol intake, glucocorticoid use, rheumatoid arthritis, secondary osteoporosis, height, and weight and can be computed with and without FN BMD. Of note, having HIV was considered in this study to be a risk factor for secondary osteoporosis.

### Ethical considerations

The study obtained ethical approvals from the Biomedical Research and Training Institute Institutional Review Board (reference: AP152/2019), the Joint Research Ethics Committee for the University of Zimbabwe College of Health Sciences and the Parirenyatwa Group of Hospitals, and the Harare Central Hospital Ethics Committee (reference: HCHEC 181119/66), as well as the Medical Research Council of Zimbabwe (reference: MRCZ/A/2551). Informed written consent was collected from all participants in their preferred language (English or Shona). Participants were reimbursed for their travel to the clinic and provided with refreshments.

### Statistical analysis

Data were cleaned and analyzed using Stata 17 (StataCorp, College Station, TX, USA). All quantitative variables are summarized using the mean ± SD if normally distributed, or otherwise, as median with an interquartile range (IQR). Categorical variables are presented as frequencies with percentages. Comparisons of quantitative variables by HIV status were performed using the Student’s *t* test and the Mann-Whitney U test if normally distributed or skewed, respectively. The chi-square and Fisher’s exact tests were used to compare categorical variables (including fracture history and proportion of women with a T-score ≤ –2.5) by HIV status. Linear regression was used to determine the association between clinical risk factors used in FRAX and femoral neck BMD reporting standardized beta-coefficients and their 95% CIs (presenting SD changes in FN BMD per unit increase of the exposure). Logistic regression was used to understand the associations between clinical risk factors and (binary) prior fracture, reporting odds ratios (ORs) and the respective 95% CIs. Finally, Poisson regression was used to quantify associations between clinical risk factors and the FRAX probability of MOF. The Poisson beta-coefficients were then exponentially transformed to generate expected probabilities and 95% CIs. In addition to clinical risk factors, SES and current contraception use (Depo-Provera) were explored in univariate analysis. Multivariable models were mutually adjusted for FRAX risk factors (age, weight, height, prior fracture, parental hip fracture, current smoking, glucocorticoids, rheumatoid arthritis, HIV infection [as a secondary cause of osteoporosis], alcohol intake). A sensitivity analysis was performed excluding women who reported a hysterectomy to ensure that the menopause groups were not biased, as this had minimal impact and models with the larger *n* are presented.

## Results

### Study population

**Figure 1:**
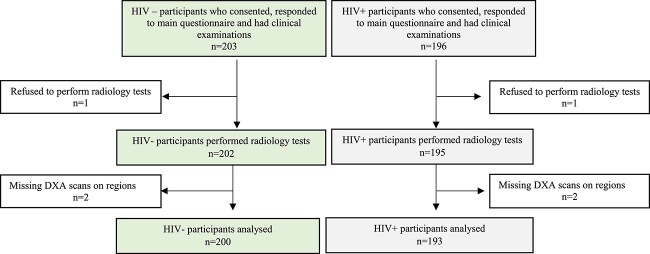
Participant recruitment flow diagram.

Of 399 women enrolled in the study, 6 (1.5%) were excluded: 2 declined to have a DXA scan and 4 had missing scans of LS and/or TH (Figure 1). We thus analyzed data from 393 (98.5%) women, of whom 193 (49.1%) were living with HIV; the overall median (IQR) age was 49 (45–54) years. The characteristics of women, stratified by HIV status, are shown in Table 1. Of the 393 women, 172 (43.8%) were postmenopausal, 141 (35.9%) were multiparous (≥4 children), and almost half were unemployed (Table 1). Twenty-one women (10 HIV-negative, 11 HIV-positive) had undergone hysterectomy (of whom 20 also had an oophorectomy). Women living with HIV had had fewer children, were less likely to currently take contraceptives (including Depo-Provera), were less likely to have attained tertiary education, were more likely to be unemployed, and were more worried about household food insecurity. Few women reported drinking alcohol (*n* = 30; 7.6%) and ever smoking (*n* = 6; 1.5%). Obesity was common, affecting 2 in 5 women, although this was more common in women who were HIV-negative (*n* = 102; 51%) compared with those with HIV (*n* = 55; 28.5%). Among women living with HIV, 95.3% (*n* = 184) were established on ART, for a median duration of 9 (5–13) years, and 84.8% (*n* = 156) were taking a regimen containing tenofovir disoproxil fumarate (TDF) (Table 1).

**Table 1:** Participant demographic, clinical, and anthropometric characteristics, by HIV status.

**Variable**	***n***	**Total (*n* = 393)**	**HIV– (*n* = 200)**	**HIV+ (*n* = 193)**	***p***
**Sociodemographic data, *n* (%)**					
**Age groups** ** 40-44 y** ** 45-49 y** ** 50-54 y** ** 55-60 y**	393	94 (23.9) 103 (26.2) 100 (25.5) 96 (24.4)	51 (25.5) 49 (24.5) 50 (25.0) 50 (25.0)	43 (22.3) 54 (28.0) 50 (25.9) 46 (23.8)	.810
**Socioeconomic status quintiles** ** Q1** ** Q2** ** Q3** ** Q4** ** Q5**	393	81 (20.6) 77 (19.6) 79 (20.1) 79 (20.1) 77 (19.6)	36 (18.0) 42 (21.0) 39 (19.5) 36 (18.0) 47 (23.5)	45 (23.3) 35 (18.1) 40 (20.7) 43 (22.3) 30 (15.5)	.208
**Menopause status** ** Pre and peri** ** Post**	393	221 (56.2) 172 (43.8)	119 (59.5) 81 (40.5)	102 (52.8) 91 (47.2)	.184
**Parity** ** Nulliparous (0)** ** Low parity (1-3)** ** Multiparous (≥4)**	393	10 (2.5) 242 (61.6) 141 (35.9)	2 (1.0) 117 (58.5) 81 (40.5)	8 (4.1) 125 (64.8) 60 (31.1)	.032
**Hysterectomy**	393	21 (5.3)	10 (5.0)	11 (5.7)	.825
**Current contraception use**	393	111 (28.4)	66 (33.0)	45 (23.3)	.033
**Current Depo-Provera use**	393	25 (6.4)	16 (8.0)	9 (4.7)	.040
**Highest level of education** ** None/primary** ** Secondary** ** Tertiary**	393	62 (15.8) 267 (67.9) 64 (16.3)	35 (17.5) 117 (58.5) 48 (24.0)	27 (14.0) 150 (77.7) 16 (8.3)	<.001
**Currently employed**	393	202 (51.4)	133 (66.5)	69 (35.8)	<.001
**Worry of food insecurity in the household**	393	271 (69.0)	126 (63.0)	145 (75.1)	.009
**FRAX risk factors, *n* (%)**					
**Ever smoked**	393	6 (1.5)	1 (0.5)	5 (2.6)	.116
**Current alcohol intake**	393	30 (7.6)	14 (7.0)	16 (8.3)	.706
**Parental hip fracture**	393	33 (8.4)	15 (7.5)	18 (9.3)	.514
**Fracture history, *n* (%)**					
**Ever fractured**	393	41 (7.0)	14 (7.0)	27 (14.0)	.023
**Hip fracture**	393	3 (0.8)	0	3 (1.5)	.117
**Major osteoporotic fracture**	393	19 (4.8)	5 (2.5)	14 (7.3)	.028
**Fragility fracture**	393	25 (6.4)	8 (4.0)	17 (8.8)	.051
**FRAX probability**					
**Ten-year major osteoporotic fragility fracture, median (IQR)**	393	1.1 (0.9-1.6)	1.0 (0.9-1.5)	1.2 (0.9-1.8)	.001
**Anthropometry**					
**Weight, mean (SD), kg**	393	75.7 (16.3)	80.8 (16.1)	70.3 (14.8)	<.001
**Height, mean (SD), cm**	393	161.5 (5.6)	162.0 (5.4)	160.9 (5.8)	.044
**BMI (kg/m^2^), *n* (%)** ** Underweight (<18.5)** ** Normal (18.5 < 25)** ** Overweight (25 < 30)** ** Obese (≥30)**	393	6 (1.5) 95 (24.2) 135 (34.3) 157 (40.0)	0 35 (17.5) 63 (31.5) 102 (51.0)	6 (3.1) 60 (31.1) 72 (37.3) 55 (28.5)	<.001
**HIV characteristics**					
**Established on ART, *n* (%)**	193	184 (95.3)	—	184 (95.3)	
**Established on TDF-based ART regimen, *n* (%)**	184	156 (84.8)	—	156 (84.8)	
**ART duration, median (IQR), y**	184	9 (5-13)	—	9 (5-13)	
**Current HIV viral load (≥50 copies/mL), *n* (%)**	193	37 (19.2)	—	37 (19.2)	

All continuous variables are described as mean (SD), unless otherwise stated; all categorical variables are presented as a frequency (%). Twenty-one (5.3%) of the women had a hysterectomy.

Abbreviations: ART, antiretroviral therapy; TDF, tenofovir disoproxil fumarate.

### Prevalence of osteoporosis

**Figure 2:**
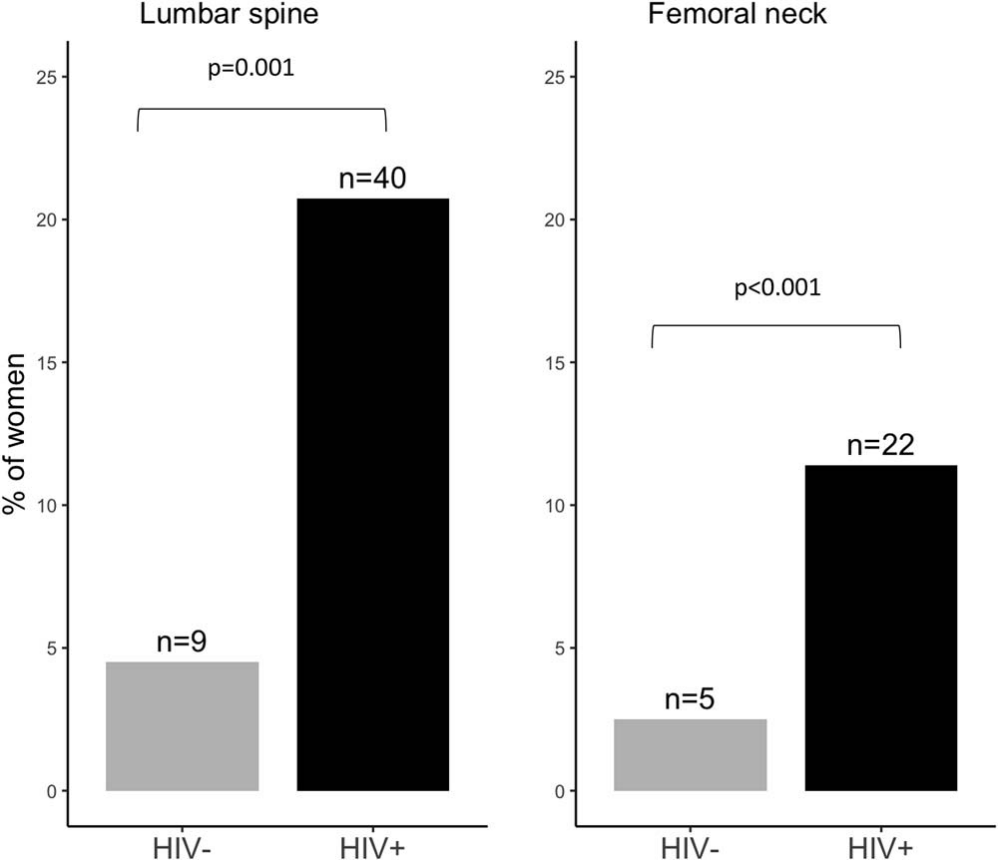
The proportion of women with a low BMD T-score (≤ –2.5) by HIV status. BMD T-scores were calculated using NHANES III reference values. Overall, 58 (21.9%) of women had osteoporosis at either the LS (49/58), FN (27/58), or TH (5/58).

**Figure 3:**
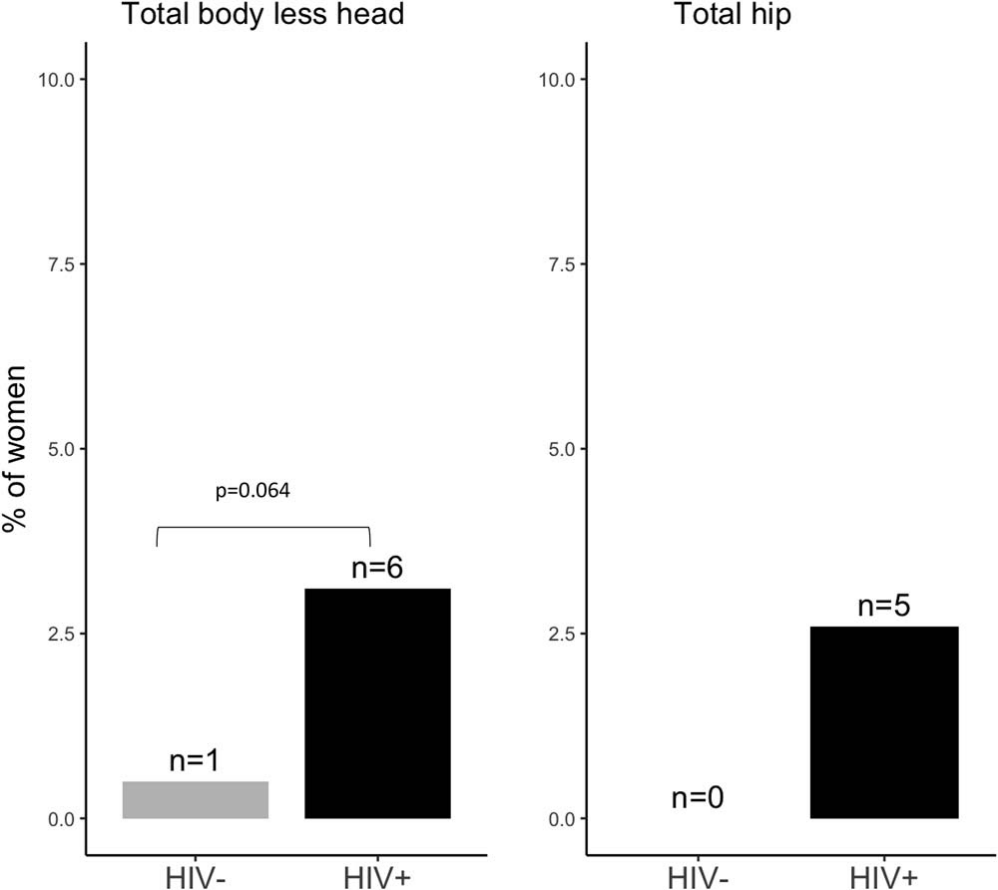
The proportion of women with a low total body and TH BMD T-score (≤ –2.5) by HIV status.

Across all participants, a T-score ≤ –2.5 was most seen at the LS (*n* = 49; 12.5%) and FN (*n* = 27; 6.9%) and less for the TH (*n* = 5; 1.3%) (Figures 1–3). However, the proportion of women with a T-score ≤ –2.5 was much higher in those living with HIV than in those without: LS (20.7% vs 4.5%), FN (11.4% vs 2.5%), and TH (2.6% vs 0). Consistently, BMD (g/cm^2^) was lower at the LS, FN, and TH in women living with HIV compared with those without (Table S1). Overall, 14.8% (*n* = 58) of women had a T-score ≤ –2.5 at either the LS, FN, or TH and those living with HIV were more commonly affected (*n* = 47; 24.3%) than those without (*n* = 11; 5.5%) (Table S2). No woman reported ever being prescribed calcium, vitamin D, or an antiresorptive or anabolic agent to reduce fracture risk.

### Prevalence of prior fracture and FRAX probability of an MOF

Overall, 10.4% (*n* = 41) of women reported a prior fracture: notably 27 (14%) women living with HIV had reported a prior fracture, compared with 14 (7%) HIV-negative women. This pattern was similar for prior MOF (14 [7.3%] vs 5 [2.5%]) and fragility fracture (17 [8.8%] vs 8 [4%]). Furthermore, women living with HIV had a higher 10-year probability of MOF compared with those without HIV, although absolute probabilities were very low (Table 1). The 10-year probability of MOF in those women who had already experienced a prior fragility fracture (*n* = 25; MOF probability: 2.2%; IQR: 1.9%–2.8%) was higher than in those women who did not report a prior fragility fracture (MOF probability: 1%; IQR: 0.8%–1.4%).

### The association between clinical risk factors and FN BMD

In univariable linear regression analysis, older age, HIV infection, and prior fracture were all associated with lower FN BMD, while greater weight and height were associated with higher FN BMD. There were no associations between lifestyle factors (smoking and alcohol intake, which were uncommon), SES, or current injectable contraceptive use (eg, Depo-Provera) with FN BMD. Although numbers were few, the mean FN BMD T-score in the 25 (6.4%) women who had experienced a prior fragility fracture was 0.08 (SD = 0.28), compared to 0.07 (SD = 0.25) in those who had not (*P*=.818).

After mutually adjusting for clinical risk factors (age, weight, height, smoking history, alcohol intake, HIV infection [as secondary cause of osteoporosis], parental hip fracture, prior fracture), every 1-year increase in age was associated with a 0.048-SD (95% CI: 0.035, 0.062) lower FN BMD. Each kilogram gain in weight was associated with a 0.031-SD (95% CI: 0.026, 0.036) higher FN BMD. Notably, women living with HIV had a 0.278-SD (95% CI: 0.115, 0.441) lower FN BMD when compared with those who were HIV negative (Table 2). Similar associations were observed between clinical risk factors and absolute FN BMD (g/cm^2^) (Table S3).

**Table 2:** Linear regression analysis for the association between FRAX risk factors and FN BMD.

	**Univariable analysis**		**Multivariable analysis**	
	**Standardized coefficient [95% CI]**	***p***	**Standardized coefficient [95% CI]**	***p***
**Age (per 1-year)**	−0.051 [−0.067, −0.035]	<.001	−0.048 [−0.062, −0.035]	<.001
**Weight (kg)**	0.035 [0.030, 0.040]	<.001	0.031 [0.026, 0.036]	<.001
**Height (cm)**	0.034 [0.017, 0.052]	<.001	−0.001 [−0.015, 0.014]	.964
**Ever smoked**	−0.663 [−1.470, 0.144]	.107	−0.265 [−0.920, 0.389]	.426
**Current alcohol intake**	0.303 [−0.069, 0.676]	.110	0.039 [-0.267, 0.345]	.802
**HIV infection**	−0.621 [−0.809, −0.432]	<.001	−0.278 [−0.441, −0.115]	.001
**Parental hip fracture**	0.217 [−0.141, 0.574]	.234	0.068 [−0.211, 0.347]	.633
**Prior fracture**	−0.329 [−0.652, −0.006]	.046	−0.150 [−0.403, 0.103]	.244

Standardized coefficients are presented, reflecting the SD change in BMD per unit increase in exposure.

### The association between clinical risk factors and prior fracture

In unadjusted analyses, women living with HIV were more likely to have had a prior fracture (OR: 2.16: 95% CI: 1.10, 4.26); a 1-SD higher FN T-score was associated with lower odds of prior fracture (OR: 0.75; 95% CI: 0.57, 0.99). Socioeconomic status was not associated with history of prior fracture. These results were attenuated after adjusting for the other clinical risk factors. After mutual adjustment, no association was observed between age, weight, height, or lifestyle factors with history of prior fracture (Table 3).

**Table 3:** Logistic regression analysis for the association between FRAX risk factors and prior fracture.

	**Univariable analysis**		**Multivariable analysis**	
	**Odds ratio [95% CI]**	***p***	**Odds ratio [95% CI]**	***p***
**Age (per 1-year)**	1.05 [0.99, 1.11]	.095	1.04 [0.98, 1.11]	.198
**Weight (kg)**	0.99 [0.97, 1.01]	.473	1.01 [0.98, 1.03]	.801
**Height (cm)**	1.01 [0.95, 1.06]	.891	1.02 [0.96, 1.08]	.489
**Ever smoked**	—		—	
**Current alcohol intake**	1.35 [0.45, 4.10]	.590	1.23 [0.39, 3.94]	.722
**HIV infection**	2.16 [1.10, 4.26]	.026	1.98 [0.95, 4.12]	.068
**FN T-score**	0.75 [0.57, 0.99]	.047	0.81 [0.56, 1.17]	.270
**Parental hip fracture**	2.06 [0.80, 5.34]	.135	1.99 [0.74, 5.36]	.173

BMD T-scores were calculated using the NHANES III reference values. All participants on Depo-Provera reported no fractures. The socioeconomic status (SES) coefficient represents the SD change in outcome for each quintile increase in SES.

Abbreviation: NHANES, National Health and Nutrition Examination Survey.

### The association between clinical risk factors and the 10-year probability of MOF

In the univariable Poisson regression model, older age, HIV infection, parental hip fracture, and prior fracture were each associated with an increase of at least 3% in 10-year probability of an MOF. Greater weight and FN T-score were independently associated with lower predicted MOF probability (Table 4). Socioeconomic status or Depo-Provera use were not associated with 10-year probability of an MOF in univariate analysis. In multivariable analyses, including all clinical risk factors, for each year of age, women had a 2% (95% CI: 1%, 3%) greater 10-year MOF probability. For every 1-unit (SD) increase in FN T-score the 10-year probability of an MOF was 14% (95% CI: 6%, 21%) lower. A history of parental hip fracture and prior fracture were both associated with higher 10-year probabilities of MOF of 2.1% (95% CI: 1.7%, 2.7%) and 1.8% (95% CI: 1.4%, 2.2%), respectively. However, no independent association was seen between HIV infection (as a cause of secondary osteoporosis) and 10-year MOF probability (Table 4).

**Table 4:** Poisson regression analysis for the association between FRAX risk factors and 10-year probability of major osteoporotic fracture.

	**Univariable analysis**		**Multivariable analysis**	
	**10-Year probability** **[95% CI]**	***p***	**10-Year probability** **[95% CI]**	***p***
**Age (per 1-year)**	1.03 [1.02, 1.04]	<.001	1.02 [1.01, 1.03]	.045
**Weight (kg)**	0.96 [0.95, 0.99]	.018	0.99 [0.97, 1.01]	.888
**Height (cm)**	0.99 [0.98, 1.01]	.269	0.99 [0.98, 1.01]	.863
**Ever smoked**	1.18 [0.85, 1.65]	.594	1.14 [0.58, 2.22]	.710
**Current alcohol intake**	1.20 [0.90, 1.62]	.224	1.14 [0.83, 1.57]	.427
**HIV infection**	1.26 [1.14, 1.41]	.007	1.03 [0.86, 1.25]	.703
**FN T-score**	0.46 [0.44, 0.57]	<.001	0.86 [0.79, 0.94]	.001
**Parental hip fracture**	2.18 [1.91, 2.46]	<.001	2.12 [1.66, 2.66]	<.001
**Prior fracture**	2.07 [1.82, 2.36]	<.001	1.79 [1.43, 2.22]	<.001

The expected probabilities are presented as rate = e^β^; with the β coefficients calculated from the Poisson models. BMD T-scores were calculated using the NHANES III reference values. FRAX calculation included HIV as a secondary cause for osteoporosis. The socioeconomic status (SES) coefficient represents the SD change in outcome for each quintile increase in SES.

Abbreviation: NHANES, National Health and Nutrition Examination Survey

## Discussion

In a population of urban-dwelling Zimbabwean women aged 40-60 years living with HIV and an HIV-negative comparison group, we have described osteoporosis prevalence and explored the potential utility of the recently calibrated proxy FRAX tool for Zimbabwe. Despite the relatively young age range, BMD T-score–defined osteoporosis was more common among women with HIV (24%) than among HIV-negative women (5.5%). Osteoporosis was observed at important skeletal sites at risk of fragility fracture, such as the LS and FN. Consistently, women with HIV were also more likely than their uninfected peers to have sustained a previous fracture (both any fragility fracture and MOF). Older age, lower weight, and HIV infection were found to be strongly associated with low FN BMD, while the proxy FRAX tool–predicted 10-year probability of MOF appeared to be driven by some different risk factors, including prior fracture and parental history of hip fracture. Ten-year MOF risk, as estimated by the Zimbabwean FRAX tool, calibrated using data from Black South Africans,^15^ returned a lower-than-expected fracture risk for both groups, even in those with a prior fragility fracture, suggesting that it may underestimate fracture risk. Notably, no woman with osteoporosis or a prior fracture had been prescribed any medication to reduce fracture risk.

### Comparisons of osteoporosis prevalence with other African and global populations

While data on osteoporosis prevalence, particularly using DXA T-score definitions, are limited in southern Africa, recently published cross-country comparisons have suggested that prevalence may be similar to that of White (American) women and higher than in the US Black population.^30^ Early estimates of prevalence were based on heel quantitative ultrasound (QUS), which is not recommended for use in clinical practice.^31^ Studies from West Africa reporting QUS-derived measures placed the prevalence of osteoporosis in women aged over 50 years at 17.9% in Cameroon^32^ (*n* = 212) and 18.2% in Nigeria^33^ (*n* = 110). However, neither publication clearly states the exact reference population against which T-scores were computed, making comparisons difficult. A recent study using a DXA-measured T-score of ≤ –2.5 (NHANES III reference standard, White female, aged 20–29 years) reported an estimated osteoporosis prevalence of 26.4% in rural and urban Kenyan postmenopausal women (*n* = 254, aged 50 to 95 years).^34^ In South Africa, where HIV affects 23.5% of adult women (aged 15–49 years), osteoporosis prevalence has been reported from both rural and urban settings.^14^^,^^35^ Among 258 rural-dwelling women aged 50–80 years, 37% of those living with HIV had an FN and 20% an LS T-score ≤ –2.5 (based on the White reference NHANES III), while the prevalence was lower in those without HIV, at 16% and 18%, respectively.^35^ In urban Soweto, among 205 women aged ≥50 years, osteoporosis was most common at the LS, with a prevalence of 16% (25% for women with HIV vs 15% for those without) compared with the TH affecting 5% (10% for women with HIV vs 1.6% for women without; all compared with reference ranges in NHANES III of White women). Overall, the prevalence of osteoporosis at either the LS or the TH was 25.0% and 15.7% in women living with HIV and HIV-negative women, respectively. Hence, the current osteoporosis prevalence of 24% in mid-aged women living with HIV in Zimbabwe is higher than previously reported in this region, given their younger age, than previous estimates. This may, in part, be explained by the additional economic and nutritional challenges in Zimbabwe and/or the greater challenges to universal health coverage than seen elsewhere in the region.^36^^,^^37^ For example, no woman with osteoporosis or a prior fracture had been prescribed any medication to reduce fracture risk. Until the World Health Organization (WHO) adds estrogen replacement and antiresorptive therapies (which are all available at low cost) to its essential medicine list,^38^ medication availability and usage are unlikely to change.

### Comparison of self-reported fracture rates

Women living with HIV consistently had experienced twice the proportion of prior fractures and major osteoporotic and fragility fractures compared with women living without HIV. Data on the prevalence and/or incidence of fractures across sub-Saharan Africa are few, and while there have been efforts to estimate the global burden of fractures across the region,^39^ the validity of estimates is questionable given the underlying lack of data. The only published prospective fragility fracture incidence study has come from South Africa and showed higher than previously appreciated hip fracture incidence rates among Black South African men and women, who make up the majority of the ethnically diverse population^15^; however, incidence was not studied by HIV status. A recent single-center prospective incidence study from Ethiopia reported that 25% of women compared to only 5% of men with musculoskeletal trauma had an osteoporotic fracture, defined by the authors as a fall from standing height or less in those aged 40 years or older. Of those, hip fractures made up 59.9% of the MOFs and 5.9% of all fractures.^40^ Retrospective data from Botswana, collected by patient chart review, have also been published, with rates lower than neighboring South Africa.^41^

Studies of fracture prevalence in people living with HIV have almost exclusively focused on high-income countries, where HIV is predominantly seen in men.^33^^,^^34^ Female cohorts are few^36^^,^^37^ and, in general, are younger with different sociodemographic risk factors (eg, intravenous drug use) from those in the present study. While at baseline in the Women’s Interagency HIV Study in the United States (mean age: 40 [8.8] years) there was little difference in fracture incidence rates by HIV status,^36^ after 10 years’ follow-up, women living with HIV had a higher adjusted fracture rate than HIV-uninfected women.^37^ Unadjusted incidence rates of fracture at any skeletal site were higher in women with HIV (2.19/100 person-years) compared with those without (1.54/100 person-years).^37^

### The utility of the proxy FRAX tool in Zimbabwe

As of 2018, FRAX models were available for 64 countries, the majority of which were high-income countries, although with increasing middle-income country representation.^42^^,^^43^ It is important to note that the FRAX calculations are based on clinical risk factors with weighting derived from high-income-country–based research. For example, in the Zimbabwean context, parental hip fracture may not have the same predictive value as in the United States, as life expectancy in Zimbabwe is much lower.^44^ FRAX has been developed in high-income settings, where the heritable component of fragility fracture risk is manifest through parental longevity; however, in a low-resource setting with greater environmental challenges (eg, double burden of malnutrition, greater risk of intercurrent infection), the relative risk ascribed to parental fracture history may exceed that observed in a population with few surviving parents. Notably, in the current study, the FRAX probabilities for 40–60-year-old Zimbabwean women were much lower than in neighboring South Africa, despite using the same hip fracture incidence data, likely reflecting the (comparably high) competing risk of death in Zimbabwe, used to calibrate FRAX; 2019 mean life expectancy in males was 57.5 vs 62.2 years and in females was 63.6 vs 68.3 years in Zimbabwe and South Africa, respectively.^45^

HIV was strongly associated with both FN BMD and FRAX probability of MOF, with weak evidence of an independent association with prior fracture; however, when taking account of prior fracture, parental hip fracture, and FN T-score in determining the probability of MOF, the addition of HIV as a secondary cause of osteoporosis added nothing to fracture risk prediction. This may be explained if FN BMD mediates all of the effect of HIV infection on fracture risk; however, an independent effect on (1) bone microarchitecture and strength^13^^,^^46^^,^^47^ and (2) falls risk^48^ seems likely, and therefore, FRAX may underestimate fracture risk in people living with HIV, as has been reported in high-income settings.^17^^,^^49^ It seems likely that fracture risk assessment approaches in this setting need to give greater weight to environmental challenges manifesting as malnutrition plus long-term HIV and its treatment, and place less emphasis on family history; ultimately, longitudinal studies with fracture outcomes are needed to refine and validate clinical approaches.

### Strengths and limitations

These are unique data captured from an underrepresented population, which have some generalizability to the wider southern African region where the prevalence of HIV in women at midlife is likely increasing due to aging of HIV cohorts. This analysis is strengthened by the incorporation of rarely available fracture data, which allowed an understanding of associations between clinical risk factors and prior fracture in this population. However, the study has limitations. The study design is cross-sectional, so temporal directions of association cannot be established. Because of pandemic-associated lockdown restrictions, recruitment identified those without HIV by snowballing, potentially introducing selection bias. Fracture history was captured by self-report and is subject to recall bias. We also lacked participant-reported lactation history, although in this setting where breastfeeding is ubiquitous, it is likely that this would be colinear with parity and, as such, we would not have been able to include both in the multivariable models. We also did not collect information on calcium and vitamin D intake. Unfortunately, it was not logistically possible to measure bone turnover markers or biochemistry related to calcium-phosphate metabolism in this setting in 2020. This was largely due to logistics of maintaining temperature control and laboratory facilities where basic amenities are challenging, and to overseas shipping restrictions. We prioritized measurement of viral load over CD4 count, limiting our ability to use CD4-based HIV staging. Finally, the Zimbabwean FRAX tool used is a proxy, having been calibrated using South African hip fracture incidence data, and women likely differ from those in the Zimbabwean population (eg, body composition, food insecurity).

In conclusion, findings suggest that Zimbabwean women aged 40-60 years and living with HIV have a high and previously unrecognized prevalence of osteoporosis at clinically important skeletal sites. Consistent with this, prior fracture, including fragility fracture, was approximately twice that seen in women without HIV. No women reported use of any medication to reduce fracture risk. Despite the high prevalence of osteoporosis and prior fracture, FRAX-predicted 10-year probabilities of MOF seemed disproportionately low, suggesting that context-specific adaption and interpretation is needed.

## Supplementary Material

ASBMR-24020140_Supplementary_Material_2024_07_29_zjae138

## Data Availability

The data used to prepare this manuscript are available from the senior author upon reasonable request.
