# Supplementary material for: Prevalence of HIV-associated osteoporosis and fracture risk in midlife women: a cross-sectional study in Zimbabwe
Source: J Bone Miner Res. 2024 Jul 9;39(10):1464–73. doi: 10.1093/jbmr/zjae138 (PMC11425699; doi:10.1093/jbmr/zjae138)

**Supplementary material HIV-associated osteoporosis and fracture risk in menopausal Zimbabwean women**

**Supplemental table 1: Comparison of absolute BMD by HIV status**

| **Variable** | **n** | **Total**  **(n=393)** | **HIV –**  **(n=200)** | **HIV +**  **(n=193)** | **p value** |
| --- | --- | --- | --- | --- | --- |
| TB-LH | 393 | 0.996 (0.10) | 1.025 (0.10) | 0.966 (0.10) | <0.001 |
| LS (L1-L4) | 393 | 0.987 (0.16) | 1.035 (0.15) | 0.935 (0.16) | <0.001 |
| FN | 393 | 0.880 (0.14) | 0.923 (0.14) | 0.835 (0.13) | <0.001 |
| TH | 393 | 0.980 (0.17) | 1.020 (0.15) | 0.937 (0.18) | <0.001 |

*TB-LH: Total body less-head; LS: Lumbar spine; FN: Femoral neck; TH: Total hip; BMD is measured in g/cm^2^*

**Supplemental table 2: Comparison of the prevalence of osteoporosis by HIV status**

| **Variable** | **n** | **Total**  **(n=393)** | **HIV - (n=200)** | **HIV + (n=193)** | **p value** |
| --- | --- | --- | --- | --- | --- |
| TB-LH, n (%)  ≥ -1  -1< -2.5  ≤ -2.5 | 393 | 334 (85.0)  52 (13.2)  7 (1.8) | 186 (93.0)  13 (6.5)  1 (0.5) | 148 (76.7)  39 (20.2)  6 (3.1) | <0.001 |
| LS, n (%)  ≥ -1  -1< -2.5  ≤ -2.5 | 393 | 223 (56.7)  121 (30.8)  49 (12.5) | 139 (69.5)  52 (26.0)  9 (4.5) | 84 (43.5)  69 (35.7)  40 (20.8) | <0.001 |
| FN, n (%)  ≥ -1  -1< -2.5  ≤ -2.5 | 393 | 210 (53.4)  156 (39.7)  27 (6.9) | 132 (66.0)  63 (31.5)  5(2.5) | 78 (40.4)  93 (48.2)  22 (11.4) | <0.001 |
| TH, n (%)  ≥ -1  -1< -2.5  ≤ -2.5 | 393 | 312 (79.4)  76 (19.3)  5 (1.2) | 177 (88.5)  23 (11.5)  0 | 135 (69.9)  53 (27.5)  5 (2.6) | <0.001 |
| Osteoporosis at either LS, TH or FN, n (%) | 196 | 43 (21.9) | 9 (9.0) | 34 (35.4) | <0.001 |

*TB: Total body; TB-LH: Total body less head; LS: Lumbar spine; FN: Femoral neck. All continuous variables are described as mean (SD), else stated; BMD: Bone Mineral Density; BMD T-scores*

**Supplemental table 3: Linear regression analysis for the association between FRAX-related factors and femoral neck BMD (g/cm^2^)**

|  | **Univariable analysis** | | **Multivariable analysis** | |
| --- | --- | --- | --- | --- |
|  | **Beta coefficient**  **[95% CI]** | **p-value** | **Adjusted Beta coefficient [95% CI]** | **p-value** |
| Age | -0.007 [-0.010, -0.005] | <0.001 | -0.007 [-0.009, -0.005] | <0.001 |
| Weight | 0.005 [0.004, 0.006] | <0.001 | 0.004 [0.003, 0.005] | <0.001 |
| Height | 0.005 [0.002, 0.007] | <0.001 | 0.001 [-0.002, 0.002] | 0.965 |
| Ever smoked | -0.093 [-0.207, 0.020] | 0.107 | -0.038 [-0.130, 0.055] | 0.425 |
| Alcohol intake | 0.043 [-0.010, 0.096] | 0.110 | 0.005 [-0.037, 0.049] | 0.801 |
| HIV infection | -0.088 [-0.114, -0.061] | <0.001 | -0.039 [-0.062, -0.016] | <0.001 |
| Parent hip fracture | 0.031 [-0.020, 0.081] | 0.234 | 0.010 [-0.030, 0.049] | 0.638 |
| Prior fracture | -0.046 [-0.092, -0.001] | 0.046 | -0.021 [-0.057, 0.014] | 0.242 |

*Beta coefficients are presented in g/cm^2^; CI: Confidence interval; ^a^p<0.001, ^b^p<0.05,* *SES coefficient represents the SD change in outcome for each quintile increase in SES.*

**Supplemental Figure 1:** The distribution of DXA measured BMD for different sites by HIV status


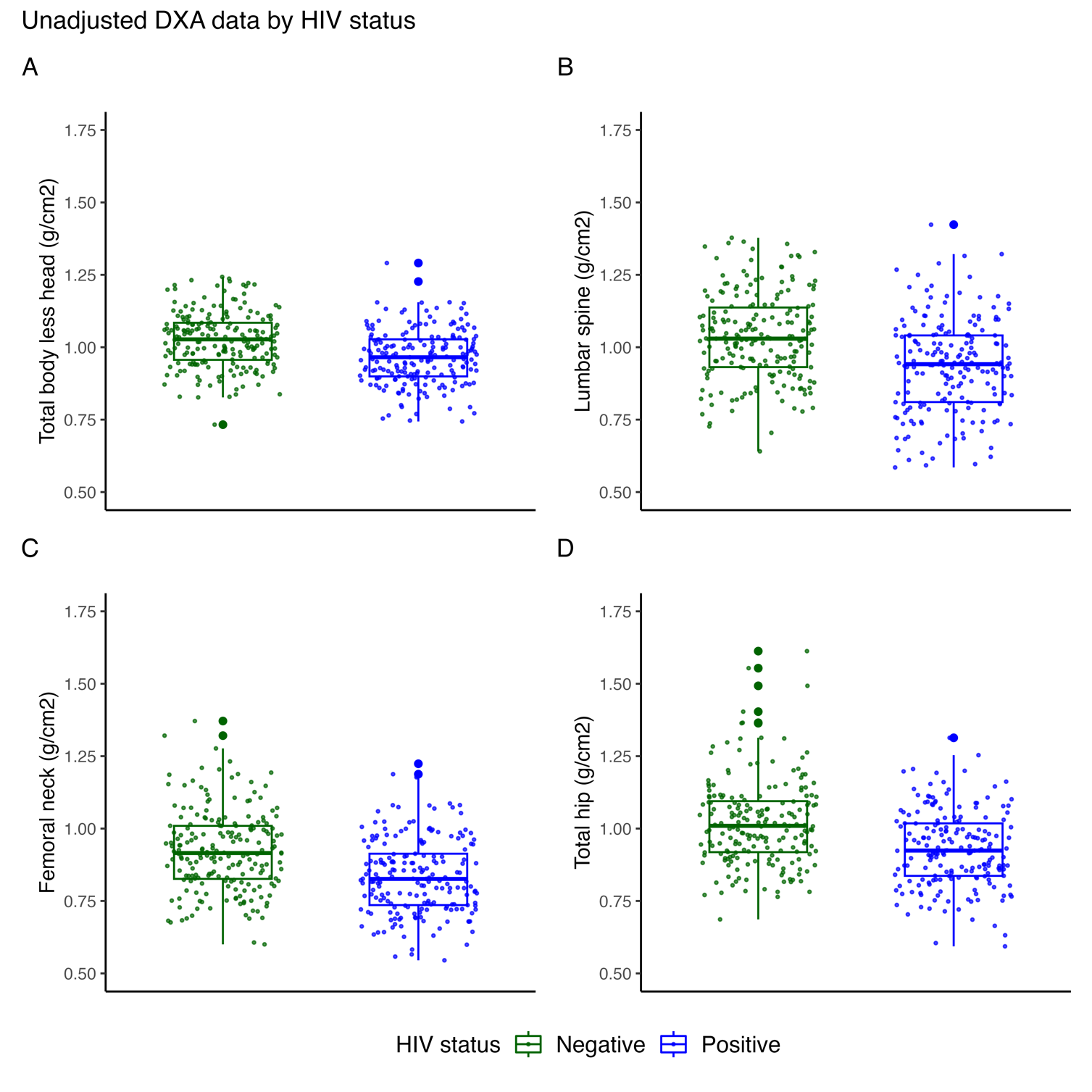

Supplement: ASBMR-24020140_Supplementary_Material_2024_07_29_zjae138 [file asbmr-24020140_supplementary_material_2024_07_29_zjae138.docx]
